# Feasibility of hippocampus-sparing VMAT for newly diagnosed glioblastoma treated by chemoradiation: pattern of failure analysis

**DOI:** 10.1186/s13014-020-01552-0

**Published:** 2020-05-06

**Authors:** Chan Woo Wee, Kyung Su Kim, Chae-Yong Kim, Jung Ho Han, Yu Jung Kim, In Ah Kim

**Affiliations:** 1 Department of Radiation Oncology, Seoul National University Bundang Hospital, Seongnam-si, Republic of Korea; 2 Department of Neurosurgery, Seoul National University Bundang Hospital, Seongnam-si, Republic of Korea; 3 Department of Internal Medicine, Seoul National University Bundang Hospital, Seongnam-si, Republic of Korea; 4 Department of Radiation Oncology, Seoul National University College of Medicine, Seoul, Republic of Korea; 5 Cancer Research Institute, Seoul National University College of Medicine, Seoul, Republic of Korea

**Keywords:** Glioblastoma, Volumetric-modulated arc therapy, Hippocampus, Hippocampus-sparing radiotherapy, Pattern of failure

## Abstract

**Background:**

To identify the pattern of failure and oncological safety of hippocampus (HC)-sparing IMRT (HSRT) in newly diagnosed glioblastoma (GBM) patients.

**Materials and methods:**

Eighty-two GBM patients treated with temozolomide-based chemoradiation using HSRT between 2014 and 2018 were retrospectively reviewed. HSRT consisted of a sparing of D_max_ of the contralateral HC < 17 Gy. Fifteen patients were unable to achieve the dose-constraints for adequate target coverage. The dose to ipsilateral HC was kept as low as possible. The pattern of failure was investigated, focusing on the area in the vicinity of the spared HC (organ and + 1 cm area). The median HSRT dose was 60 Gy in 30 fractions.

**Results:**

The median follow-up for survivors was 11.7 months. The median progression-free and overall survival were 9.7 and 23.5 months, respectively. Six (7.3%) and eight (9.8%) patients eventually demonstrated progressive disease at the contralateral HC and HC + 1 cm, respectively. The 12-month contralateral HC and HC + 1 cm failure-free rate were 97.2 and 93.4%, respectively. However, no patient (0%) and two patients (2.4%) showed failure at contralateral HC and HC + 1 cm at initial progression, respectively. The dominant pattern of failure at the contralateral HC was by subependymal seeding (66.7%).

**Conclusion:**

The incidence of failure at the contralateral HC and HC + 1 cm is very low and mostly accompanied by disseminated disease progression after HSRT. Since HSRT does not compromise oncological outcomes, it could be considered especially for GBM patients who are expected to have favorable survival outcomes.

## Introduction

Glioblastoma (GBM), the most common brain cancer in adults, is treated by radiotherapy (RT) plus concurrent and adjuvant temozolomide as first-line treatment in fit patients [1]. Despite the high recurrence rate and the dismal survival of most GBM patients, patients with gross total removal, O^6^-methylguanine-DNA methyltransferase (*MGMT)* promoter methylation, and/or isocitrate dehydrogenase (*IDH*) mutation tend to show longer survival [2].

Brain RT is well-known to be related with deterioration of neurocognitive functions. In particular, due to the association between the hippocampal neural stem cells and memory function, irradiation of the hippocampus (HC) results in decline of the cognitive and memory functions [3–5]. In the treatment of brain metastasis, hippocampus-sparing whole brain RT was proven to be effective in the preserving verbal memory function in a recent clinical trial [6]. Hippocampus-sparing RT (HSRT) was also evaluated in primary brain tumors, using sophisticated RT techniques, especially intensity-modulated RT [7–17]. We have previously reported that contralateral HC (cHC) can be effectively spared in patients with primary brain tumors via volumetric-modulated arc therapy (VMAT) to preserve the verbal memory function [18].

However, due to the diffuse infiltrative nature of GBM, the oncological safety regarding the risk of recurrence in spared HC should be validated. Here, we report the first report focusing on the progression rate in the cHC region to ensure the oncological safety of RT with maximal sparing of the cHC (cHSRT) for GBM.

## Materials and methods

### Study design

The current study is a retrospective single institutional study reviewing the clinical outcomes of GBM patients prospectively treated by cHSRT according to medical records and magnetic resonance imaging (MRI) findings. The Institutional Review Board of the Seoul National University Bundang Hospital (IRB No. B-1909/562–101) approved our study.

### Patients

Between 2014 and 2018, a total of 82 newly diagnosed GBM patients were treated with cHSRT-based chemoradiation at Seoul National University Bundang Hospital. All patients were pathologically confirmed as GBM by either surgery or biopsy. The clinical demographics of patients are displayed in Table 1.

**Table 1:** Patient and tumor characteristics

Variables	Number	Percent
Total	82	(100.0)
Median age (years, range)	57.7	(24.0–86.0)
Sex		
Male	49	(59.8)
Female	33	(40.2)
KPS		
90–100	48	(58.5)
70–80	24	(29.3)
< 70	10	(12.2)
Molecular profiles		
*MGMT* promoter methylation		
Yes	37	(45.1)
No	45	(54.9)
*IDH1* mutation		
Yes	4	(4.9)
No	78	(95.1)
Surgery		
GTR	34	(41.5)
PR	33	(40.2)
Biopsy	15	(18.3)
Location		
Right	43	(52.4)
Left	33	(40.2)
Bilateral	6	(7.3)
Subventricular zone involvement		
Yes	50	(61.0)
No	32	(39.0)
Spatial relationship to the subventricular zone [19]		
Group I	34	(41.5)
Group II	15	(18.3)
Group III	28	(34.1)
Group IV	5	(6.1)
Median PTV volume^a^ (cc, range)	304.1	(85.1–648.9)

*Abbreviations*: *KPS* Karnofsky Performance Score, *MGMT* methylated O^6^-methylguanine-DNA methyltransferase, *IDH1* mutated isocitrate dehydrogenase 1, *GTR* gross total resection, *PR* partial resection, *PTV* planning target volume

^a^The volume of initial field for patients using a shrinking field technique

### Radiotherapy

All patients were treated by temozolomide-based chemoradiation using VMAT following surgery or biopsy according to the Stupp regimen (1). In most patients (75/82, 91.5%) the prescribed RT dose was 60 Gy in 30 fractions. Two patients were treated by 56 Gy in 28 fractions due to the proximity to the optic chiasm/nerve(s), and five patients were treated by hypofractionated RT (40.5–48 Gy in 15–18 fractions) due to poor performance, multifocal lesions, or old age. The RT planning computed tomography (CT) scans were acquired by a Brilliance CT Big Bore™ CT simulator (Philips, Cleveland, OH, USA) in 2-mm thickness. All CT images were fused with 1-mm thickness T1-enhanced and T2 fluid attenuated inversion recovery images from postoperative MRI. All contours were delineated by 1 of the 2 radiation oncologists (I.A.K. and C.W.W.) and peer-reviewed by both during the current study. Delineation of the HC was performed following the RTOG protocols [20]. The clinical target volume was delineated by using a 1.0–2.0-cm margin around the gross tumor volume and the resection cavity per clinicians’ preference.

**Fig. 1:**
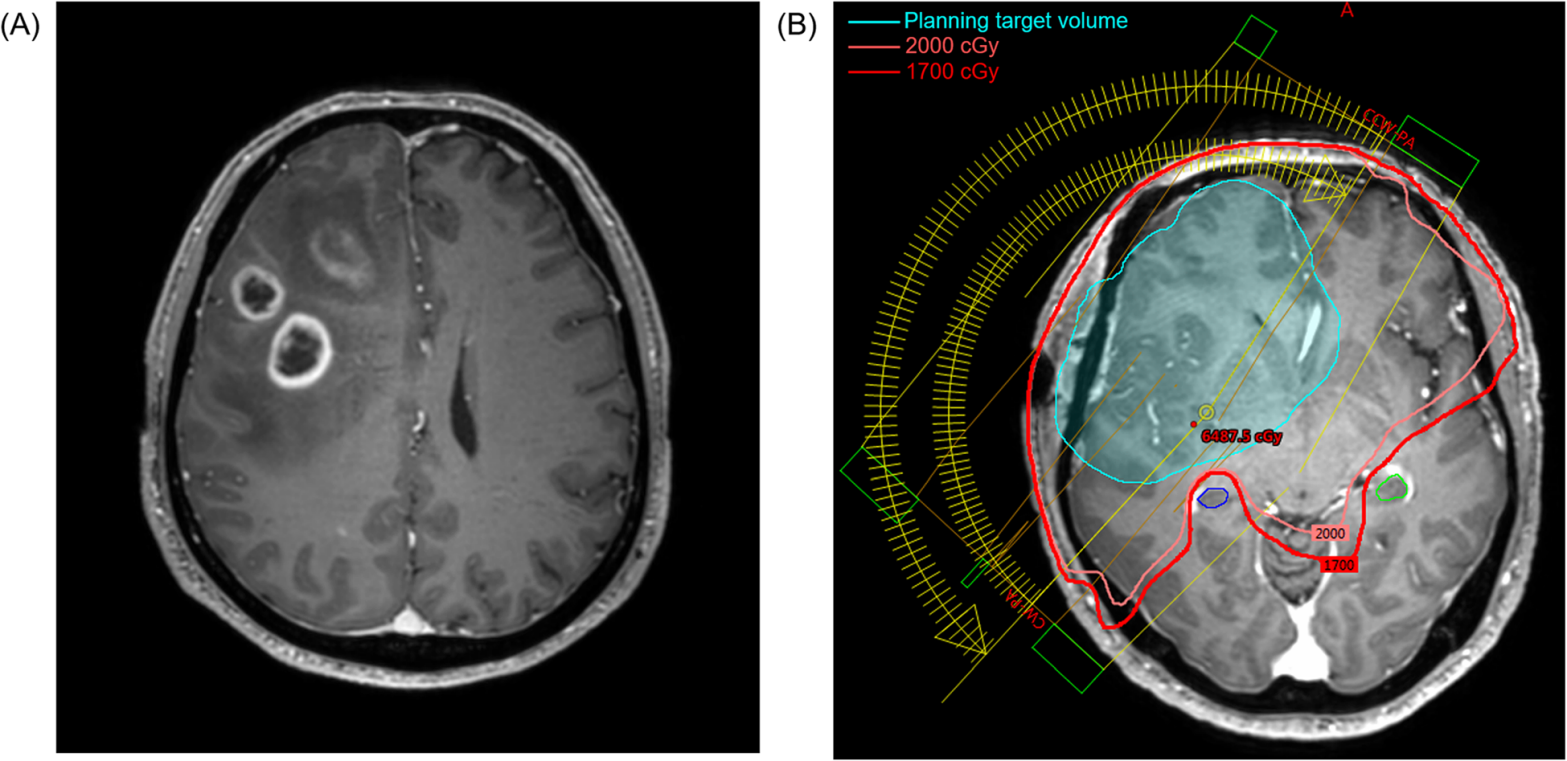
A 65-year old man showing multiple enhancing lesions involving the right frontal lobe, corpus callosum body/splenium, and right parietal lobe on (**a**) preoperative magnetic resonance imaging. The patient was pathologically confirmed as *IDH1* wild-type glioblastoma with methylated *MGMT* promoter after receiving partial resection of the tumor followed by temozolomide-based chemoradiotherapy of 60 Gy in 30 fractions. **b** Using 2 partial arcs, the maximum dose to the right (ipsilateral, blue contour) and left (contralateral, green line) hippocampi were 16.93 Gy and 16.38 Gy, respectively, on a volumetric-modulated radiotherapy plan. Abbreviations: *IDH1*, Isocitrate dehydrogenase 1; *MGMT*, O^6^-methylguanine-DNA methyltransferase

The dose constraint for the cHC was set at a maximum dose (D_max_) of less than 17 Gy without compromising the target coverage (Fig. 1). However, in cases where the planning target volume (PTV) encroached the cHC, the dose was kept as low as possible. Efforts to obtain the lowest dose as possible for ipsilateral HC was made unless it was not included in the PTV. Constraints for the brain stem and optic chiasm/nerve(s) was set according to the QUANTEC guideline. The mean dose (D_mean_) of the HC as an equivalent dose in 2-Gy fraction was calculated assuming alpha-beta ratio of 2 Gy (EQD_2/2_). All RT plans were reviewed in the Eclipse treatment planning system ver. 13.7 (Varian Medical Systems, Palo Alto, CA, USA) for dosimetric analysis.

### Follow-up

Every patient was monitored by a multidisciplinary neuro-oncology team composed of a radiation oncologist, neurosurgeon, medical oncologist, radiologist, and pathologist. The first follow-up brain MRI was performed at post-chemoradiation 4 weeks. For 2 years, follow-up MRI was done at least every 3 months.

To evaluate the cHC-recurrence, we evaluated the actual involvement of the spared cHC itself by disease as well as the involvement of the area with a 1-cm margin around the cHC (cHC + 1 cm) in every follow-up MRIs. To identify the risk factors for cHC- and cHC + 1 cm-failure, a univariate analysis was done for variables of the following: *MGMT* promoter methylation status, *IDH1* mutational status, surgery type, subventricular zone involvement, and spatial relationship to the subventricular zone of the primary enhancing tumor according to Lim et al. [19].

In 49 patients (49/82, 59.8%) whose whole ipsilateral HC (iHC) was not included in the PTV, and therefore were eligible sparing at least a partial volume of the iHC, we evaluated the probability of iHC-failure rate as well.

### Statistical analysis

Survival and time to progression was calculated from the date of surgery or biopsy to the date of last follow-up or event. Progression-free survival was defined as disease progression or death. All statistical analysis was done by using the Statistical Package for Social Sciences, version 23.0 (IBM Corp., Armonk, NY, USA). Level of statistical significance was set at a *P*-value under 0.05.

## Results

### Dosimetric analysis

The median dose to 100% volume (D_100%_) and D_max_ of the cHC were 6.71 Gy and 16.10 Gy, respectively. However, 15 patients (18.3%) could not fulfill the dose constraint of the cHC with D_max_ higher than 17 Gy (range, 17.02–52.75 Gy). The median D_mean_ expressed in EQD_2/2_ to the cHC was 6.59 Gy_2_. The median D_max_ and D_mean_ (EQD_2/2_) of the evaluated ipsilateral HC were 46.69 Gy and 13.22 Gy_2_, respectively. The median D_max_ to the brain stem and optic chiasm were 51.77 Gy and 36.6 Gy, respectively. Other organs at risk could be effectively spared as well. The detailed results of the dosimetric analysis can be found in Table 2.

**Table 2:** Dosimetric analysis of organs at risk

Organs at risk	Dose (Gy)	Median	interquartile range		
Hippocampus					
Contralateral	Vol (cc)	1.70	1.40	–	2.15
	D_100%_	6.71	2.96	–	9.70
	D_max_	16.10	13.30	–	16.85
	D_mean_	10.94	7.59	–	12.58
	D_mean_ (EQD_2/2_)	6.59	4.27	–	7.61
Ipsilateral^a^	Vol (cc)	1.50	1.05	–	1.80
	D_100%_	8.44	2.66	–	16.69
	D_max_	46.69	16.70	–	57.52
	D_mean_	19.70	8.54	–	38.86
	D_mean_ (EQD_2/2_)	13.22	4.89	–	32.62
Bilateral^a^	D_mean_	15.15	8.31	–	25.01
	D_mean_ (EQD_2/2_)	9.59	4.75	–	18.44
Optic chiasm	D_max_	36.60	18.85	–	50.70
Optic nerve	D_max_	14.90	2.37	–	46.99
Brain stem	D_max_	51.77	22.40	–	59.54
Eyeballs	D_max_	6.18	1.99	–	21.81
Lenses	D_max_	2.08	0.93	–	5.19

*Abbreviations*: *EQD*_*2/2*_ equivalent dose in 2-Gy fraction assuming alpha-beta ratio of 2 Gy

^a^Excluded for analysis if whole ipsilateral hippocampus was included within the planning target volume

### Survival and failure at hippocampi

**Fig. 2:**
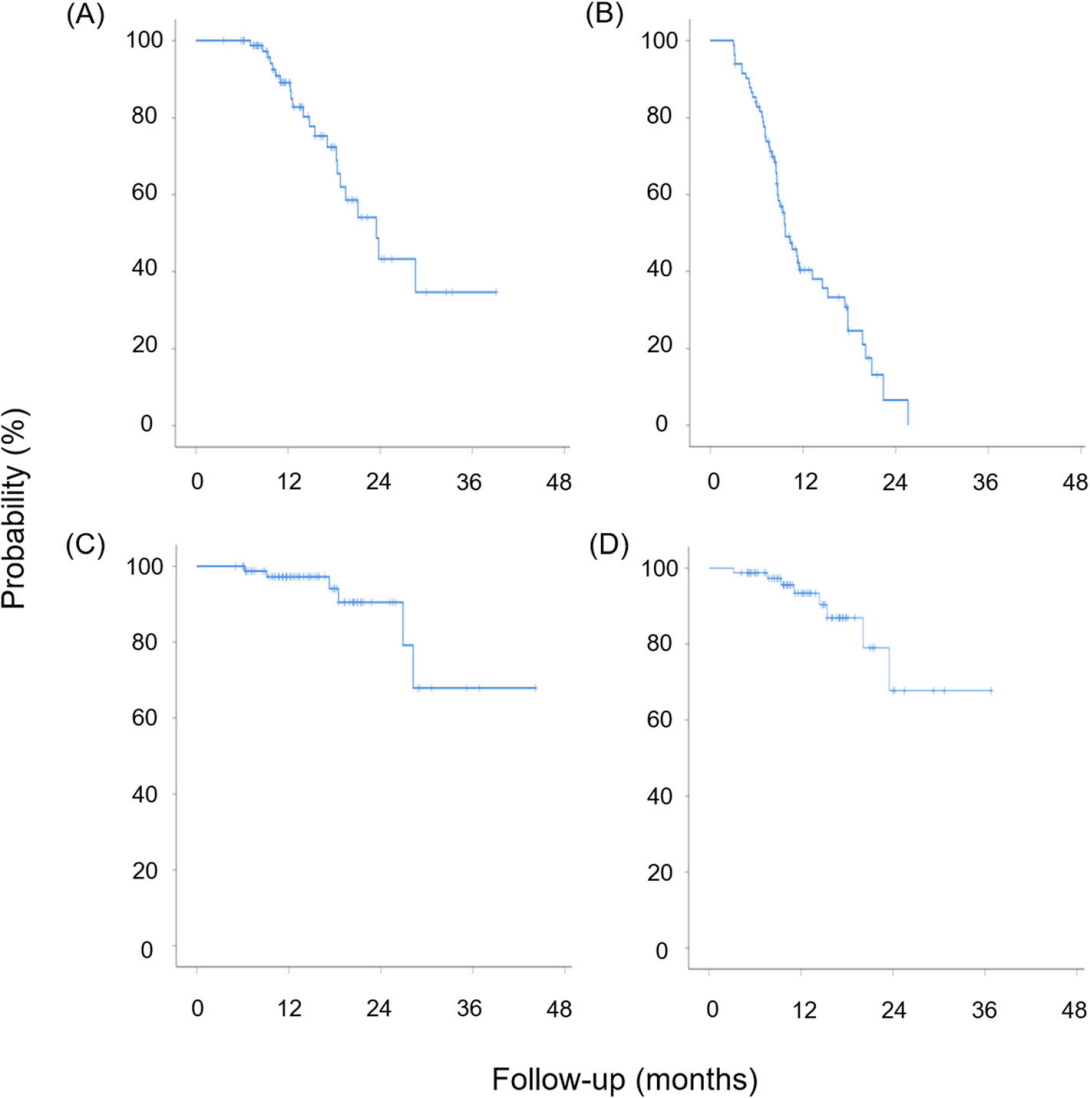
The Kaplan-Meier curves of (**a**) overall survival, (**b**) progression-free survival, (**c**) contralateral hippocampus failure-free rate, and (**d**) contralateral hippocampus+ 1 cm failure-free rate in all patients (*n* = 82)

The median follow-up for survivors was 11.7 months (range, 3.6–39.1). The median overall and progression-free survival were 23.5 (95% confidence interval, 18.4–28.7) (Fig. 2a) and 9.7 (95% confidence interval, 7.9–11.5) months (Fig. 2b) by Kaplan-Meier survival analysis, respectively.

In respect of the tumor-failure at the cHC, 6 (6/82, 7.3%) and 8 (8/82, 9.8%) patients eventually demonstrated disease progression at the cHC and cHC + 1 cm, respectively. The 6-month and 12-month cHC failure-free rate were 98.7 and 97.2%, respectively (Fig. 2c). The rate of 6-month and 12-month failure-free rate at cHC + 1 cm were also high with 98.8 and 93.4%, respectively (Fig. 2d). No patient (0/82, 0%) and only 2 patients (2/82, 2.4%) showed tumor-failure at the cHC and cHC + 1 cm as initial disease progression, respectively. In the patients with disease-failure at cHC or cHC + 1 cm, the median interval from initial disease progression was 5.0 and 4.2 months, respectively. The dominant pattern of failure at the cHC and cHC + 1 cm were by subependymal seeding rather than direct tumor infiltration with rates of 66.7% (4/6) and 50.0% (4/8), respectively.

Of the 6 patients with cHC-failure, 4 patients died during follow-up. The median interval between cHC-failure and death due to disease was 1.4 months (range, 0.1–5.1 months). Follow-up of the remaining 2 patients after cHC-failure was 0.2 and 2.4 months. Similarly, in the 8 patients with failure at cHC + 1 cm, 6 patients died after a median interval of 1.4 months (range, 0.1–5.1 months).

In the univariate analysis investigating the risk factors for failure at cHC and cHC + 1 cm, only subventricular zone involvement of the primary enhancing tumor was a significant risk factor for cHC-failure (*P* = 0.034) (Table 3). The 12-month cHC failure-free rate was 95.5 and 100.0% for patients with and without subventricular zone involvement, respectively. However, it was not a significant factor for cHC + 1 cm failure (*P* = 0.113). Methylation of the *MGMT* promoter, mutation of the *IDH1* gene, extent of surgery, or spatial relationship to the subventricular zone classified in 4 groups [19] did not affect the failure rate at cHC nor cHC + 1 cm (Table 3).

**Table 3:** Univariate analysis for contralateral hippocampus failure

Variables	cHC failure-free rate^a^		*P*^*b*^	cHC + 1 cm failure-free rate^a^		*P*^*b*^
	6 months (%)	12 months (%)		6 months (%)	12 months (%)	
All patients	98.7 ± 1.3	97.2 ± 1.9		98.8 ± 1.2	93.4 ± 3.3	
*MGMT* promoter methylation			0.868			0.628
Yes	100.0	100.0		100.0	92.4 ± 5.1	
No	97.6 ± 2.4	94.7 ± 3.6		97.8 ± 2.2	94.9 ± 3.5	
*IDH1* mutation			0.080			0.234
Yes	100.0	100.0		100.0	100.0	
No	98.6 ± 1.3	97.1 ± 2.0		98.7 ± 1.3	93.0 ± 3.5	
Surgery			0.594			0.790
GTR	97.1 ± 2.9	97.1 ± 2.9		97.1 ± 2.9	92.8 ± 5.0	
PR	100.0	100.0		100.0	95.5 ± 4.4	
Biopsy	100.0	91.7 ± 8.0		100.0	91.7 ± 8.0	
Subventricular zone involvement			0.034			0.113
Yes	97.9 ± 2.1	95.5 ± 3.1		98.0 ± 2.0	91.8 ± 4.8	
No	100.0	100.0		100.0	95.7 ± 4.3	
Spatial relationship to the subventricular zone [19]			0.196			0.211
Group I	97.1 ± 2.9	93.8 ± 4.2		97.1 ± 2.9	88.3 ± 6.7	
Group II	100.0	100.0		100.0	100.0	
Group III	100.0	100.0		100.0	100.0	
Group IV	100.0	100.0		100.0	75.0 ± 21.7	

*Abbreviations*: *cHC* contralateral hippocampus, *MGMT* methylated O6-methylguanine-DNA methyltransferase, *IDH1* mutated isocitrate dehydrogenase 1, *GTR* gross total resection, *PR* partial resection

^a^Failure rates are displayed with standard errors

^b^Log-rank test

Regarding the iHC-failure in evaluated 49 patients, 12 (24.5%) eventually demonstrated failure during follow-up. The rate of 6-month and 12-month failure-free rate at iHC were 100 and 81.2%, respectively. All iHC-failures were due to local progression from the primary tumor site and beyond the PTV. Similar to patients who have failed at the cHC, patients with iHC-failure showed a poor survival of median 5.1 months after iHC-failure. Half of them (6/12) died within a median interval of 2.0 months (range, 0.1–6.0 months).

### Dose to the contralateral hippocampus and failure

We further explored whether the 67 patients who satisfied the dose-constraint of D_max_ < 17Gy (*n* = 65) showed inferior cHC- or cHC + 1 cm-failure compared to the 15 patients whose cHC was irradiated to higher doses. By log-rank test for cHC-failure, there was no significant difference between those 2 patient groups (*P* = 0.564). Four (4/67, 6.0%) and two (2/15, 13.3%) patients demonstrated cHC in patients with cHC-D_max_ of <17Gy and > 17Gy, respectively. There was no difference in cHC − + 1 cm-failure between the groups as well (*P* = 0.962). There were 6 (6/67, 9.0%) and 2 (2/15, 13.3%) cHC + 1 cm-failures in each group, respectively.

## Discussion

Based on this analysis of 82 patients with GBM treated with cHSRT and temozolomide, we did not observe tumor failure at the cHC at initial disease progression, with a 1-year cumulative failure rate at cHC of only 2.8%. Moreover, progression-free and overall survival were not compromised with cHSRT compared to previous studies where patients were treated with temozolomide-based chemoradiation [1, 2].

For primary brain tumors, the safety of compromising the target volume for HC-sparing is not justified. In high-grade gliomas, recurrences are most often located within 2-cm of the original tumor [21], which often makes sparing of the iHC difficult. Moreover, the American Society for Radiation Oncology guidelines for GBM noted that since published data validating the oncological safety for HC-sparing in GBM patients, the panel does not recommend compromising the target coverage for the protection of HC [22]. Hence, the purpose of our strategy was to at least spare the cHC without compromising the coverage of the PTV. We have previously reported that cHSRT can be effectively done in patients with primary brain tumors via VMAT leading to preservation of the verbal memory function [18]. However, the study included patients with heterogeneous histology. For the application of this strategy to aggressive and highly infiltrative high-grade tumors such as GBM, it is necessary to prove the safety of the strategy in respect of survival and relapse patterns. In this study, we have successfully demonstrated the low incidence of failure at the cHC as well as cHC + 1 cm confirming the oncological safety of cHSRT in GBM.

Numerous studies have assessed the association between the treatment margins and recurrence patterns in high-grade gliomas, especially GBM [21]. Typically, RT clinical target volume is delineated with a 2–3-cm margin around the T1-enhancing lesions and/or T2 signal abnormality on MRIs. However, since central/infield recurrence accounts for the majority around 80%, several groups evaluated the pattern of failure after using a reduced margin. It has been reported that reduced margins do not affect the outcome and failure patterns in GBM [21, 23–25]. Furthermore, Ali and colleagues reported that reduced RT margin results in significant dose reduction for the HC [12]. In our study, we have reduced the cHC dose without violating the recommended target volume delineation using the VMAT technique. In most clinical settings, cHC may locate exclusively outside the PTV even without reduced margins, unless the tumor crosses to the contralateral hemisphere via the corpus callosum.

Moreover, the survival after experiencing disease failure at the cHC or cHC + 1 cm was very poor with most patients dying within 5 months (median, 1.4 months), indicating that cHC-failure occurs mostly at the terminal stage of the disease. Indeed, in most of our patients with cHC, the disease was already disseminated throughout the whole cerebral hemisphere with multiple subependymal seeding lesions, and therefore proceeded to best supportive care. This finding further supports that covering the cHC in the RT field is not a primary concern, and therefore, the cHC can be safely spared. Although the iHC-failure rate seemed to be higher with a failure-free rate of 81.2% at 12 months after a median follow-up period of 11.2 months compared to cHC-failure, no patient who had their iHC spared failed until 6 months of follow-up. Furthermore, all 12 iHC-failures were a subsequent event following a local failure within the PTV, and the iHC was not the site of failure at initial recurrence. Similar to patients experiencing cHC-failure, patients with iHC-failure had a poor median survival period of 5.1 months. The patients who were alive at our last follow-up might demonstrate a similarly poor survival with a longer follow-up since the median follow-up for the alive patients with iHC failure was only 2.5 months. Therefore, according to the results from our study, sparing of the iHC, at least a partial volume, seems reasonable as long as the coverage of the PTV is not violated.

According to our effort to identify the underlying risk factors for cHC-failure, only subventricular zone involvement of the primary tumor laid patients to significantly higher risk for cHC-failure. This was probably due to the increased risk of seeding of tumor cells through the ventricular space considering that the dominant route of cHC-failure was by subependymal seeding. However, this process occurred mostly at the terminal stage of the disease, and the absolute risk of cHC-failure was still very low even in patients with subventricular zone involvement with a 6-month and 12-month cHC failure-free rate of 97.9 and 95.5%, respectively. Therefore, we concluded that cHC-sparing RT in patients with subventricular zone can also be justified.

In general, overall survival of GBM has been considered poor. However, risk stratification according to genetic profiles such as *MGMT* promoter methylation or *IDH* gene mutation of the tumor demonstrated a subgroup of patients, although a minority, shows an unexpected long-term survival for GBM [2, 26]. Hence, HSRT could be considered in those GBM patients to maintain cognitive and memory functions. This strategy may also be extended to the treatment of less aggressive brain tumors. However, the association between the integral dose to the normal brain tissue occurring due to arc therapy and the long-term neurocognitive changes should be carefully investigated in low-grade tumors, especially in younger patients. Further studies should clarify the benefit of HSRT in partial brain RT.

This study has several limitations such as the retrospective nature of the study. Moreover, since pre- and post-cHSRT neurocognitive function test was not performed in all patients, the adequate dose-constraint to the cHC as well as the dose-response between neurocognitive decline and irradiated dose of the cHC in GBM patients cannot be identified by the current study. Nevertheless, since previous studies have demonstrated an overt association between irradiation of the HC and cognitive decline [3–5], the authors believe that the very first study presenting the oncological safety of cHSRT in GBM patients is of value.

## Conclusion

In summary, given the low incidence of cHC- and cHC + 1 cm-failure, chemoradiation with cHSRT seems to be safe in newly diagnosed GBM as far as the target volume is not compromised.

## Data Availability

All data generated or analyzed during this study are included in this published article and its supplementary information files. Any eventual further details on the data related to this study, are available from the corresponding author on reasonable request.

## References

[1] Stupp R, Mason WP, van den Bent MJ, Weller M, Fisher B, Taphoorn MJB (2005). Radiotherapy plus concomitant and adjuvant Temozolomide for Glioblastoma. N Engl J Med.

[2] Wee CW, Kim E, Kim N, Kim IA, Kim TM, Kim YJ (2017). Novel recursive partitioning analysis classification for newly diagnosed glioblastoma: a multi-institutional study highlighting the MGMT promoter methylation and IDH1 gene mutation status. Radiother Oncol.

[3] Gondi V, Tomé WA, Mehta MP (2010). Why avoid the hippocampus? A comprehensive review. Radiother Oncol.

[4] Kazda T, Jancalek R, Pospisil P, Sevela O, Prochazka T, Vrzal M (2014). Why and how to spare the hippocampus during brain radiotherapy: the developing role of hippocampal avoidance in cranial radiotherapy. Radiat Oncol.

[5] Monje ML, Toda H, Palmer TD (2003). Inflammatory blockade restores adult hippocampal neurogenesis. Science..

[6] Gondi V, Pugh SL, Tome WA, Caine C, Corn B, Kanner A (2014). Preservation of memory with conformal avoidance of the hippocampal neural stem-cell compartment during whole-brain radiotherapy for brain metastases (RTOG 0933): a phase II multi-institutional trial. J Clin Oncol.

[7] Marsh JC, Godbole R, Diaz AZ, Gielda BT, Turian JV (2011). Sparing of the hippocampus, limbic circuit and neural stem cell compartment during partial brain radiotherapy for glioma: a dosimetric feasibility study. J Med Imaging Radiat Oncol.

[8] Marsh J, Godbole R, Diaz A, Herskovic A, Turian J (2012). Feasibility of cognitive sparing approaches in children with intracranial tumors requiring partial brain radiotherapy: a dosimetric study using tomotherapy. J Can Res Ther.

[9] Panet-Raymond V, Ansbacher W, Zavgorodni S, Bendorffe B, Nichol A, Truong PT (2012). Coplanar versus noncoplanar intensity-modulated radiation therapy (IMRT) and volumetric-modulated arc therapy (VMAT) treatment planning for fronto-temporal high-grade glioma. J Appl Clin Med Phys.

[10] Marsh JC, Ziel GE, Diaz AZ, Wendt JA, Gobole R, Turian JV (2013). Integral dose delivered to normal brain with conventional intensity-modulated radiotherapy (IMRT) and helical tomotherapy IMRT during partial brain radiotherapy for high-grade gliomas with and without selective sparing of the hippocampus, limbic circuit and neural stem cell compartment. J Med Imaging Radiat Oncol..

[11] Oehler J, Brachwitz T, Wendt TG, Banz N, Walther M, Wiezorek T (2013). Neural stem cell sparing by linac based intensity modulated stereotactic radiotherapy in intracranial tumors. Radiat Oncol.

[12] Ali AN, Ogunleye T, Hardy CW, Shu HK, Curran WJ, Crocker IR (2014). Improved hippocampal dose with reduced margin radiotherapy for glioblastoma multiforme. Radiat Oncol.

[13] Bodensohn R, Sohn M, Ganswindt U, Schupp G, Nachbichler SB, Schnell O (2014). Hippocampal EUD in primarily irradiated glioblastoma patients. Radiat Oncol.

[14] Pinkham MB, Bertrand KC, Olson S, Zarate D, Oram J, Pullar A (2014). Hippocampal-sparing radiotherapy: the new standard of care for World Health Organization grade II and III gliomas?. J Clin Neurosci.

[15] Canyilmaz E, Uslu GD, Colak F, Hazeral B, Haciislamoglu E, Zengin AY (2015). Comparison of dose distributions hippocampus in high grade gliomas irradiation with linac-based imrt and volumetric arc therapy: a dosimetric study. SpringerPlus..

[16] Adeberg S, Harrabi SB, Bougatf N, Bernhardt D, Rieber J, Koerber SA (2016). Intensity-modulated proton therapy, volumetric-modulated arc therapy, and 3D conformal radiotherapy in anaplastic astrocytoma and glioblastoma: a dosimetric comparison. Strahlenther Onkol.

[17] Smyth G, Evans PM, Bamber JC, Mandeville HC, Welsh LC, Saran FH (2016). Non-coplanar trajectories to improve organ at risk sparing in volumetric modulated arc therapy for primary brain tumors. Radiother Oncol.

[18] Kim KS, Wee CW, Seok J-Y, Hong JW, Chung J-B, Eom K-Y (2018). Hippocampus-sparing radiotherapy using volumetric modulated arc therapy (VMAT) to the primary brain tumor: the result of dosimetric study and neurocognitive function assessment. Radiat Oncol.

[19] Lim DA, Cha S, Mayo MC, Chen MH, Keles E, VandenBerg S (2007). Relationship of glioblastoma multiforme to neural stem cell regions predicts invasive and multifocal tumor phenotype. Neuro-Oncology.

[20] Hippocampal contouring: a contouring Atlas for RTOG 0933. http://www.rtog.org/LinkClick.aspx?fileticket=59vaU8vfgQc%3d&tabid=338. Accessed 2 Nov 2019.

[21] Wernicke AG, Smith AW, Taube S, Mehta MP (2016). Glioblastoma: radiation treatment margins, how small is large enough?. Pract Radiat Oncol..

[22] Cabrera AR, Kirkpatrick JP, Fiveash JB, Shih HA, Koay EJ, Lutz S (2016). Radiation therapy for glioblastoma: executive summary of an American Society for Radiation Oncology evidence-based clinical practice guideline. Pract Radiat Oncol..

[23] McDonald MW, Shu H-KG, Curran WJ, Crocker IR (2011). Pattern of failure after limited margin radiotherapy and temozolomide for glioblastoma. Int J Radiat Oncol Biol Phys.

[24] Paulsson AK, McMullen KP, Peiffer AM, Hinson WH, Kearns WT, Johnson AJ (2014). Limited margins using modern radiotherapy techniques does not increase marginal failure rate of glioblastoma. Am J Clin Oncol.

[25] Guram K, Smith M, Ginader T, Bodeker K, Pelland D, Pennington E (2019). Using smaller-than-standard radiation treatment margins does not change survival outcomes in patients with high-grade Gliomas. Pract Radiat Oncol.

[26] Wee CW, Kim IH, Park CK, Kim JW, Dho YS, Ohka F (2018). Validation of a novel molecular RPA classification in glioblastoma (GBM-molRPA) treated with chemoradiation: a multi-institutional collaborative study. Radiother Oncol.

